# Current and Future Applications of Artificial Intelligence in Clinical Genetics

**DOI:** 10.7759/cureus.100581

**Published:** 2026-01-01

**Authors:** Janet Lawrence

**Affiliations:** 1 Medical Education, Great Western Hospital, Swindon, GBR

**Keywords:** artificial intelligence, clinical genetics, genomics, machine learning, precision medicine, preventive medicine

## Abstract

Artificial Intelligence (AI) is transforming medicine, and its influence on clinical genetics cannot be ignored. From variant analysis and diagnostic interpretation to predictive and prognostic medicine, AI is improving the efficacy of genetic and genomic medicine using machine learning and deep learning systems to process large sequences of genomic data.

This editorial explores current applications, highlights emerging advances, and discusses briefly the potential trajectory of AI in clinical genetics and our role to play as healthcare professionals. It also touches on the ethical limitations of AI in medicine.

## Editorial

Artificial Intelligence (AI) has captured widespread attention in this era. Beyond its use in everyday activities, AI is becoming more important in the field of medicine [1]. In the field of clinical genetics, AI is expected to make substantial progress, influencing the way clinicians investigate, diagnose, and manage genetic disorders [1].

With the introduction of Next Generation Sequencing (NGS), genomic data output has increased exponentially [2]. Manually analysing, interpreting, and reporting this vast amount of data is both time-consuming and prone to human bias [3]. This highlights the need for technological tools capable of handling and interpreting complex datasets accurately and efficiently. Machine learning and deep learning have made breakthroughs in genomic interpretation, and this is only the beginning [4].

At present, one of the most impactful uses of AI in clinical genetics lies in variant analysis. Variant analysis is the process of classifying genetic variants into five categories (ranging from benign to pathogenic) using internationally recognised standards. It considers segregation data, phenotype matching, databases, scientific literature, predictive algorithms, and findings from functional studies [3]. This process, though essential, can be tedious and time-intensive [1].

Variant calling is the first step of variant analysis, involving the identification of insertions/deletions, single-nucleotide polymorphisms, and structural data. Variant calling tools, such as DeepVariant, DNAscope, and DeepTrio, have set new benchmarks for accuracy in variant detection [5]. Predictive algorithms and scoring systems have also been streamlined by tools such as Combined Annotation Dependent Depletion (CADD) [1]. However, clinical expertise remains crucial in clinical decision-making after variant analysis and the clinical and biological interaction with patients during the process, underscoring that AI should complement clinicians rather than replace them [3].

In clinical diagnostics, AI has proved invaluable through phenotype-driven systems. Tools such as Face2Gene use deep learning to identify subtle facial features linked to genetic syndromes [6]. Machine learning processes can be broadly divided into unsupervised learning, which identifies patterns; supervised learning, which makes classifications and predictions based on prior examples; and reinforcement learning, which applies reward and penalty strategies to refine performance [7]. When combined with deep learning, which can process complex datasets, these approaches can help diagnose rare genetic conditions earlier [7] and could potentially bridge the gap in regions with limited access to clinical genetics services [1].

AI applications also extend to genetic research. For instance, a study investigating the association of protein-encoding genes with developmental disorders analysed exome sequencing data and identified 285 significantly associated genes, including 28 that had not previously been robustly linked to these disorders [8]. This demonstrates AI’s capacity for continuous learning and adaptation.

AI has also shown promise in prognosis determination and predictive medicine, thereby advancing precision medicine. Precision medicine is a healthcare approach tailored to the specific needs of an individual [9]. In genetics, understanding risk factors and their impact is central to advancing personalised care [10]. AI can enhance this understanding by integrating multi-omic data, improving biomedical knowledge platforms, and enabling efficient epigenetic analysis of disease pathogenicity [11].

AI’s role in prognosis prediction is also evident in oncology. For example, a recent study used deep learning algorithms to quantify tumour morphology in two groups of colon cancer patients stratified by DNA mismatch repair status. By correlating these morphological features with clinical outcomes, the study enabled risk stratification and facilitated the prediction of cancer recurrence [12]. This approach not only improves prognostic accuracy but also provides insight into the underlying mechanisms driving disease progression.

Despite its benefits, the adoption of AI in healthcare faces key challenges. These include data privacy, lack of algorithmic transparency, and underrepresentation of diverse or rare populations in training datasets [13,14]. Without proper safeguards, data privacy and confidentiality can be compromised through unauthorised data collection, re-identification of anonymised data, or breaches exposing sensitive information. Data bias also poses an ethical limitation; AI models trained primarily on specific populations or disease types may lack generalisability, leading to diagnostic and therapeutic disparities [14]. These limitations highlight the need for inclusive and globally representative data frameworks.

Looking forward, AI could evolve from being a primarily interpretative tool to a predictive and preventive tool in the field of clinical genetics. Treatment in clinical genetics is still an emerging concept, but advances such as gene therapy and gene-silencing technologies have evolved. A recent Huntington’s disease trial using these gene silencing technologies showed up to a 75% slowing of disease progression [15]. This illustrates the transformative potential ahead. Integrating AI with large language models, advanced machine learning systems, and real-time clinical data could revolutionise clinical decision-making, early risk prediction, and personalised therapeutic development.

In conclusion, AI has already made a lasting impact on clinical genetics, enhancing speed, accuracy, and diagnostic depth. It should not be seen as a replacement for clinicians but as a collaborative partner in advancing patient care. With strong ethical oversight and inclusive data representation, AI could transform clinical genetics into a field that not only diagnoses but also cures.
